# Predicting the thermal distribution in a convective wavy fin using a novel training physics-informed neural network method

**DOI:** 10.1038/s41598-024-57772-x

**Published:** 2024-03-25

**Authors:** K. Chandan, Rania Saadeh, Ahmad Qazza, K. Karthik, R. S. Varun Kumar, R. Naveen Kumar, Umair Khan, Atef Masmoudi, M. Modather M. Abdou, Walter Ojok, Raman Kumar

**Affiliations:** 1 Department of Mathematics, Amrita School of Engineering, Amrita Vishwa Vidyapeetham, Bengaluru, Karnataka India; 2 Faculty of Science, Zarqa University, Zarqa, 13110 Jordan; 3 Department of Studies in Mathematics, Davangere University, Davangere, Karnataka 577002 India; 4 Department of Pure and Applied Mathematics, School of Mathematical Sciences, Sunway University, Petaling Jaya, 47500 Selangor Darul Ehsan Malaysia; 5 Department of Mathematics, Faculty of Science, Sakarya University, Serdivan/Sakarya, 54050 Turkey; 6 Department of Computer Science and Mathematics, Lebanese American University, Byblos, 1401 Lebanon; 7 College of Computer Science, King Khalid University, Abha, Saudi Arabia; 8 Department of Mathematics, College of Science and Humanities in Al-Kharj, Prince Sattam bin Abdulaziz University, Al-Kharj, 11942 Saudi Arabia; 9 Department of Mathematics, Faculty of Science, Aswan University, Aswan, 81528 Egypt; 10 Department of Chemistry, Faculty of Science, Muni University, P.O Box 725, Arua, Uganda; 11 Department of Mechanical Engineering, University Centre for Research and Development, Chandigarh University, Mohali, Punjab 140413 India

**Keywords:** Heat transfer, Fin, Wavy fin, Internal heat generation, Physics-informed neural networks, Energy science and technology, Mathematics and computing

## Abstract

Fins are widely used in many industrial applications, including heat exchangers. They benefit from a relatively economical design cost, are lightweight, and are quite miniature. Thus, this study investigates the influence of a wavy fin structure subjected to convective effects with internal heat generation. The thermal distribution, considered a steady condition in one dimension, is described by a unique implementation of a physics-informed neural network (PINN) as part of machine-learning intelligent strategies for analyzing heat transfer in a convective wavy fin. This novel research explores the use of PINNs to examine the effect of the nonlinearity of temperature equation and boundary conditions by altering the hyperparameters of the architecture. The non-linear ordinary differential equation (ODE) involved with heat transfer is reduced into a dimensionless form utilizing the non-dimensional variables to simplify the problem. Furthermore, Runge–Kutta Fehlberg’s fourth–fifth order (RKF-45) approach is implemented to evaluate the simplified equations numerically. To predict the wavy fin's heat transfer properties, an advanced neural network model is created without using a traditional data-driven approach, the ability to solve ODEs explicitly by incorporating a mean squared error-based loss function. The obtained results divulge that an increase in the thermal conductivity variable upsurges the thermal distribution. In contrast, a decrease in temperature profile is caused due to the augmentation in the convective-conductive variable values.

## Introduction

The transportation of thermal power (heat) via physical structures is the main focus of the mechanical engineering field of heat transfer. The methods of heat transfer may be categorized into convection, radiation, conduction, and energy transmission involving phase shifts. Due to the enormous rise in global energy consumption, improving heat transfer has become one of the most difficult tasks concerning saving energy in daily life. Liquid heat transmission rate improvements have grabbed the interest of scientists and engineers due to their vast and practical applications in medical, industrial, and technological sectors. Transport of heat through the nanoliquids has a wide range of applications in mechanical and engineering science, chemical analysis, and biological sectors such as solar water heating, polymers cooling of microchips, heat exchangers, food processing insulating materials, heat pipes, glass manufacturing, solar energy fluidizations, hydraulic breaks, and geothermal power extraction. As a result of these numerous applications, many scholars have investigated the influence of heat transfer on several flow circumstances. Prasannakumara et al.^1,2^ explored the radiative heat transmission aspects of the nanoliquid past a stretchy sheet. The convective heat transport in a dusty liquid flow past a stretching surface was deliberated by Prasannakumara et al.^3^ with the influence of magnetic force. Muhammad et al.^4^ considered the carbon nanomaterial for examining the heat transmission characteristics of the nanoliquid in the porous medium. Shashikumar et al.^5^ discussed the features of heat transmission in the microchannel flow by utilizing carbon nanotubes. Souayeh et al.^6^ inspected the heat transfer behaviour of the dusty nanofluid with the impact of thermal radiation. The impact of convective heating on the radiative heat transmission of fluid was debriefed by Madhu et al.^7^ and Shashikumar et al.^8^ using an inclined microchannel. Riasat et al.^9^ considered the magnetic field's effect to analyze the nanofluid flow's heat transmission aspects. Shashikumar et al.^10^ and Madhu et al.^11,12^ investigated the role of magnetic force on the heat transfer mechanism and fluid flowing through microchannel. Shashikumar et al.^13^ debriefed the influence of the convective heating boundary on the flow and heat transmission of the liquid in the microchannel. The transmission and enhancement heat of the nanofluid was examined by considering its flow over stretching and microchannel geometries and presenting the mathematical description^14–17^. Recently, the aspects of radiative heat transport of the fluid through a microchannel were analyzed by Shashikumar et al.^18^ and Madhu et al.^19^. Ramesh et al.^20^ delineated the heat transfer analysis of the biomagnetic hybrid nanoliquid over a porous thin needle. Alsulami et al.^21^ performed the heat transfer investigation of the non-Newtonian nanofluid flow past a porous media. Alhowaity et al.^22^ probed the heat transport of nanoliquid flowing past a moveable sheet using non-Fourier model. In recent years, several authors have analyzed the heat transmission of the fluid flow with the aspects of thermophoretic deposition, magnetic field, heat sink/source, and thermal radiation^23–32^. The investigation of the thermal efficacy of fins as a passive strategy for improving heat dissipation from heated main surfaces is currently receiving a lot of interest in the scientific community. The use of fins increases the effective heat transfer area and decreases the base surface temperature for a specific thermal load and heat transfer coefficient. Fins are widely exploited in some manufacturing applications, including air conditioning, oil transportation pipelines, and the cooling of computer processors. Madhura et al.^33^ inspected the temperature performance of the permeable extended surface with the radiation impact. The consequence of the surface emissivity on the convective fin was studied by Abukhaled and Khuri^34^ with variable thermal conductivity. Sarwe and Kulkarni^35^ deliberated on the temperature performance of the annular extended surface with variable thermal conductivity. Wang and Shi^36^ probed the heat transmission in the convective extended surface with variable thermal conductivity. Utilizing the collocation approach, Kumar et al.^37^ deliberated the heat transport analysis in the semi-spherical extended surface with variable thermal conductivity.

Surface shape may also aid in lowering airside resistance in addition to increasing fin surface, and wavy fins are one of the most popular techniques to increase the airside. Wavy fins improve heat transfer efficiency by prolonging the flow route, strengthening the heat transmission surface area, and creating efficient corrugations that cause vortices to mix with the cooling air. The continuous fin shape of wavy fins allows the consistent operation to withstand unfavourable ambient circumstances, even if the heat transmission of wavy fins is inferior to interrupted surfaces like louvre or slit fins. Liu et al.^38^ debriefed the improvement of heat transmission in wavy fin heat exchangers used in cooling industries. Thermohydraulic attributes of a wavy extended surface and tube exchange system with concave generators were inspected by Song et al.^39^. The heat transmission attributes of the porous wavy fin were investigated by Kumar et al.^40^ using the machine learning scheme. Sharma et al.^41^ investigated the concentrated tube-type heat storage system with wavy fin patterns. An artificial neural network scheme was employed by Kumar et al.^42^ to study thermal distribution and heat transport in a wavy fin. Internal heat is produced in the system during conduction in many engineering applications. For instance, heat is generated when electricity flows from an electric line. Heat is produced in nuclear reactors as a result of neutron absorption. Many industrial applications, including cylinders of air-cooled aircraft, jet engines, electronic products, etc., use fins in unstable state conditions. The fins of electronic equipment can generate heat. Heat generation inside nuclear reactors with fins is feasible. Investigating heat generation in fins is vital due to their diverse applications. Das and Kundu^43^ inspected the impact of heat generation on the radial permeable fin. The thermal characteristics of the convective porous fin with internal heat production were scrutinized by Din et al.^44^. The heat transmission attributes of the permeable extended surface involving internal heat generation were analyzed by Venkitesh and Mallick^45^. The influence of heat generation and variable thermal conductivity on the radiative fin was evaluated by Kaur and Singh^46^. The sumudu transform approach was employed by Gireesha et al.^47^ to study the analysis of heat transmission in a permeable extended surface. A new paradigm recognized as physics-informed neural networks (PINNs) is emerging in the relationship between physics and artificial intelligence. It is a scenario in which machines can understand the physical laws that govern a system and reproduce the data. This innovative strategy offers a glimpse into the future of artificial intelligence and has the potential to transform engineering design and scientific research. The Stiff-PINN, a neural network inspired by physics for addressing stiff chemical kinetics, was introduced by Ji et al.^48^. It examines the difficulties in applying PINN to stiff ODE systems and how the quasi-steady-state assumption can help lessen stiffness and make PINN successful. The performance of conventional PINN and Stiff-PINN in solving two classical stiff kinetic systems is contrasted in this work. This also discusses a few difficulties encountered while carrying out this research that may serve as a roadmap for future advancements. PINN is presented as a representation to mimic COVID-19 infection and hospitalization scenarios by Berkhahn and Ehrhardt^49^. The incorporation of vaccination rates and increasing transmissibility of SARS-CoV-2 and its variations using an expanded susceptible-infected-recovered (SIR) model. The PINN uses a data-driven approach with an ODE system built to account for physical laws and calculate the transport rate based on data currently accessible in Germany. Chiu et al.^50^ proposed a novel technique termed CAN-PINN, a quick neural network based on coupled-automatic-numerical differentiation. To ensure that results adhere to the laws of physics, the authors present unique approaches for practical training with increased precision. To speed up PINN training, they offer complementary numerical differentiation principles and connect nearby support points. Bararnia and Esmaeilpour^51^ used physics-based neural networks to resolve viscous and thermal boundary layer-related problems. Models are built and trained using TensorFlow, and results are compared to those obtained using finite difference methods. The study successfully employs the models to assess boundary layer thicknesses and emphasizes the influence of Prandtl number and boundary placements on necessary network complexity.

Heat transfer is crucial for various manufacturing and scientific engineering, electronic cooling systems, and various industrial processes demanding precise thermal management. Additionally, incorporating various-shaped fins in heat exchanger operations has become more prevalent due to its cost-effective and excellent performance in strengthening heat transmission. The uniqueness of using physics-informed neural networks (PINN) in the context of a wavy fin heat transfer analysis comes from its ability to combine machine learning power with domain-specific physics knowledge seamlessly. The advantages of conventional physics-based modelling are combined with neural networks' adaptability and learning potential in PINN. The essential principles guiding thermal transfer in wavy fin are preserved while allowing for a more rigorous, data-driven analysis. PINN maintains some transparency in contrast to conventional black-box machine learning models, making it simple to comprehend the underlying mechanisms and factors by providing interpretable insights into the physical processes involved in heat transmission within wavy fin. Also, very few investigations deal with wavy fins^52–55^, and considerably fewer address internal heat generation in wavy fin structures. An extensive study of the effects of internal heat generation on wavy fin structures is required, considering that these structures transport heat more efficiently than rectangular fin designs. Motivated by these significant factors, the present study examines the thermal variation in the convective wavy fin with consequences of internal heat generation. Further, the developed energy equation involving the laws governed by physics is solved by implementing the PINN. The applied technique is a novel direction in describing the solution for the proposed fin model. This technique is validated by comparing the obtained results with numerical outcomes using graphs and tables.

The proposed study provides significant insights regarding the:

- Convective heat transfer rates and dynamic thermal fluctuations in a wavy fin.
- Influence of internal heat generation on the thermal variation of the wavy fin.
- Solution of the developed heat transfer model with the application of PINN.
- Precision of the applied PINN in solving the heat transfer nonlinear equations.

## Formulation of the problem

The wavy fin of rectangular shape (WFRS) with length $L$, and width $W$ is taken for the thermal analysis. The subsequent deductions have been taken into consideration to develop the mathematical representation of the current conceptual theory:

- The fin is presumed to transmit heat with the surrounding environment having conatant temperature $T_{a}$ through convection.
- Transfer of heat from the extended surface to the adjacent fluid occurs in a steady state.
- The thermal conductivity, internal heat generation and convective heat transfer coefficient are supposed to be temperature-dependent.
- The x-axis is regarded along the overall length of the fin, initially at its primary surface.
- The heat resistance at the point of interacting between the fin and the wall is insignificant.
- The temperature of the adjacent fluid $T_{a}$, and the fin-base temperature $T_{b}$ are taken to be constants.
- Since the fin thickness is small in regard to the remaining measurements, the temperature distribution throughout the fin can be approximated as a function of only the length coordinate.

**Figure 1:**
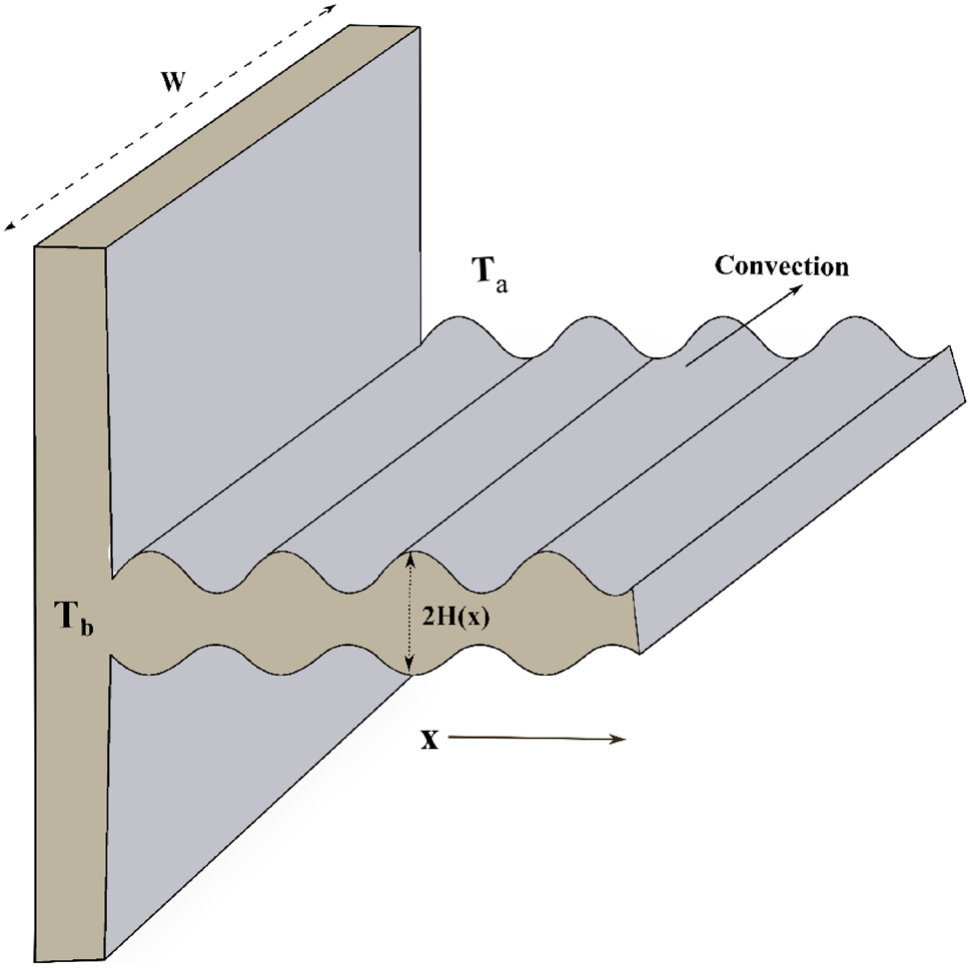
Geometrical depiction of a wavy fin.

The features of the considered model are schematically illustrated in Fig. 1.

The fin surface has been wavy along its longitudinal axis. The wavy fin structure is designed in the shape of a sine curve with (Prakash et al.^52^)

1 $$H = H_{0} \left\{ {1 + \delta \sin \left[ {2\pi n\left( \frac{x}{L} \right) + \varphi } \right]} \right\},$$

and the cross-sectional area is specified by (Poornima et al.^53^)

2 $${\rm A}_{CS} = 2H_{0} \int\limits_{0}^{W} {\left\{ {1 + \delta \sin \left[ {2\pi n\left( \frac{x}{L} \right) + \varphi } \right]} \right\}\,} dz.$$

Correspondingly, the fin surface area is given by (Poornima et al.^53^)

3 $${\rm A}_{SF} = 2W\,L_{ta} ,$$

where $$L_{ta} = \int\limits_{0}^{L} {\sqrt {1 + \left( {\frac{dH}{{dx}}} \right)^{2} } \,} dx$$.

Further, the thermal conductivity, internal heat generation and the convective heat transfer coefficient are mathematically denoted as

$${\rm K}(T) = k_{a} \left( {1 + \vartheta \left( {T - T_{a} } \right)} \right),$$

$$q^{*}(T) = q_{a} \left[ {1 + \nu \left( {T - T_{a} } \right)} \right],$$

4 $$h^{*} (T) = h_{b} \left( {\frac{{T - T_{a} }}{{T_{b} - T_{a} }}} \right)^{p} .$$

where $T_{b}$, and $h_{b}$ denote the base temperature of fin, and the convection heat transfer at $T_{b}$ respectively.

Considering the aforesaid-stated premises, the governing equation is expressed as follows (Poornima et al.^53^, Khaled^54^ and Ramesh et al.^55^):

5 $$\frac{d}{dx}\left[ {{\rm K}(T)\frac{dT}{{dx}}} \right] = \left[ {\frac{{d{\rm A}_{SF} }}{dx}} \right]\frac{{h^{*} \left( T \right)}}{{{\rm A}_{CS} }}\left( {T - T_{a} } \right) - \left[ {\frac{{{\rm K}(T)}}{{{\rm A}_{CS} }}\frac{{d{\rm A}_{CS} }}{dx}} \right]\frac{dT}{{dx}} - q^{*} (T).$$

With the substitution of above mathematical expressions (Eq. (4)), the following equation is obtained

6 $$\begin{aligned} & \frac{d}{dx}\left[ {k_{a} \left( {1 + \vartheta \left( {T - T_{a} } \right)} \right)\frac{dT}{{dx}}} \right] + k_{a} \left( {1 + \vartheta \left( {T - T_{a} } \right)} \right)\frac{dT}{{dx}}\left[ {\frac{1}{{{\rm A}_{CS} }}\frac{{d{\rm A}_{CS} }}{dx}} \right] \\ & \quad - \frac{1}{{{\rm A}_{CS} }}\left[ {\frac{{d{\rm A}_{SF} }}{dx}} \right]\frac{{h_{b} \left( {T - T_{a} } \right)^{p + 1} }}{{\left( {T_{b} - T_{a} } \right)^{p} }} + q_{a} \left[ {1 + \nu \left( {T - T_{a} } \right)} \right] = 0. \\ \end{aligned}$$

The governing equation (Eq. (6)) is related to left and right end boundary conditions, i.e. base and tip of the fin. The left one is the fixed boundary condition and insulated boundary condition is implemented at the right tip. These are mathematically represented as,

At fin’s base: $\,x = 0:\,T = T_{b}$,

At fin’s tip:

7 $$\,x = L:\,\frac{dT}{{dx}} = 0.$$

For the sake of simplicity and convenience, several dimensionless constraints are presented as:

$$\Theta = \frac{{T - T_{a} }}{{T_{b} - T_{a} }},\;X = \frac{x}{L},\;\beta = \vartheta \left( {T_{b} - T_{a} } \right),Nc = \frac{{h_{b} L^{2} }}{{k_{a} H_{0} }},a_{RL} = \frac{{H_{0} }}{L},$$

8 $$Q_{gen} = \frac{{q_{a} L^{2} }}{{k_{a} \left( {T_{b} - T_{a} } \right)}},\;\chi = \nu \left( {T_{b} - T_{a} } \right).$$

Utilization of the above established appropriate variables, Eq. (6) in the dimensionless arrangement is as follow

9 $$\begin{aligned} & \frac{d}{dX}\left[ {\left\{ {\left( {1 + \beta \,\Theta } \right)} \right\}\frac{d\Theta }{{dX}}} \right] + \left( {1 + \beta \,\Theta } \right)\left[ {\frac{{2\pi \delta n\cos \left( {2\pi nX + \varphi } \right)}}{{1 + \delta \sin \left( {2\pi nX + \varphi } \right)}}} \right]\frac{d\Theta }{{dX}} \\ & \quad - Nc\,\left[ {\frac{{\sqrt {1 + 4\left( {\pi a_{RL} \delta n} \right)^{2} \cos^{2} \left( {2\pi nX + \varphi } \right)} }}{{1 + \delta \sin \left( {2\pi nX} \right)}}} \right]\Theta^{p + 1} + Q_{gen} \left[ {1 + \chi \Theta } \right] = 0. \\ \end{aligned}$$

The transmuted boundary conditions are

10 $$\begin{gathered} X = 0\,\,:\,\,\,\,\,\Theta = 1, \hfill \\ X = 1\,\,:\,\,\,\Theta ^{\prime} = 0. \hfill \\ \end{gathered}$$

## Physics-informed neural networks (PINNs)

**Figure 2:**
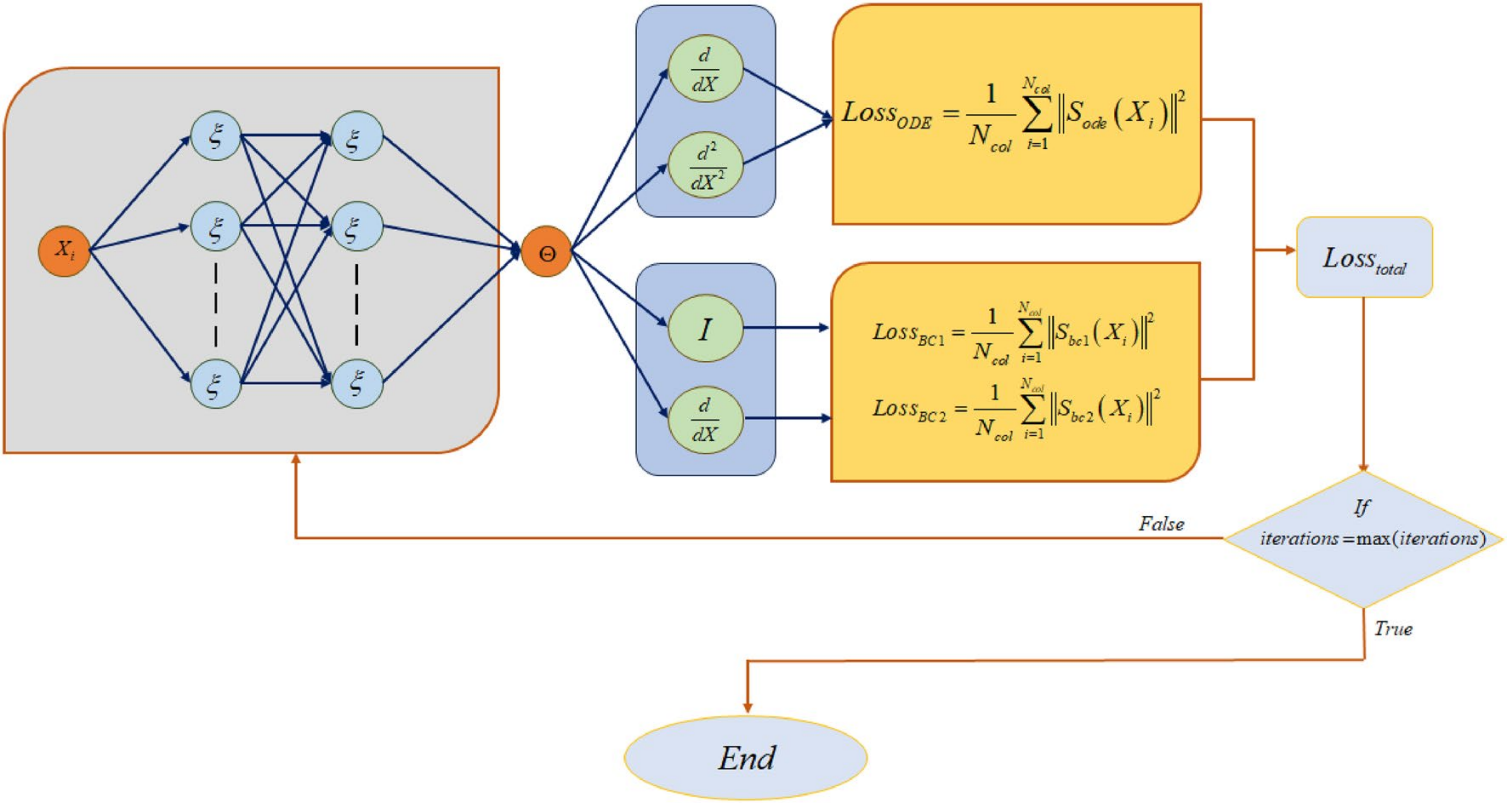
Working procedure of PINN.

Artificial neural networks have been employed in numerous technical fields, and their variety of uses keeps increasing. Neural network techniques have recently been employed by researchers to assess various physical parameters affecting heat transport mechanisms^56–59^. The development and deployment of PINNs for solving heat transfer equations (ODEs/PDEs) and similar problems represents a standard change from traditional numerical simulation-based techniques. PINN can be an effective tool for simulating scenarios with diverse boundary conditions (BCs) in close to real-time once it has been trained. This approach is instrumental in manufacturing, where process uncertainties, such as unknown BCs, necessitate speedy and reliable problem evaluation. Becoming a fascinating and crucial area of research is the potential application of densely structured neural networks as representations for resolving ODEs, notably in heat transfer. A neural network model demonstrating this concept is shown in Fig. 2. The concept of feature engineering emerges as a vital and corresponding tactic to enhance the network's ability to accurately reflect the underlying physical principles and introduce physics-guided loss functions. This methodology's careful shaping of neural network properties to closely match the numerical expression of the heat equation's solution is a crucial component. The primary layer of the neural network architecture is meticulously developed by the fusion of two preliminary layers of hidden layers to speed up the process. The first of these preparatory layers uses the positional input and activates the Rectified Linear Unit (ReLU) while applying trainable weights and biases. A careful comparison and evaluation of the effectiveness of the aforementioned architectural paradigms is done during the following validation process. Following a thorough grid search process, a layout with two hidden layers holding 62 nodes and a learning rate of 1E−04 emerges as the optimal values.

11 $$\begin{aligned} S_{ODE} = & \frac{d}{dX}\left[ {\left\{ {\left( {1 + \beta \,\Theta } \right)} \right\}\frac{d\Theta }{{dX}}} \right] + \left( {1 + \beta \,\Theta } \right)\left[ {\frac{{2\pi \delta n\cos \left( {2\pi nX + \varphi } \right)}}{{1 + \delta \sin \left( {2\pi nX + \varphi } \right)}}} \right]\frac{d\Theta }{{dX}} \\ & \quad - Nc\,\left[ {\frac{{\sqrt {1 + 4\left( {\pi a_{RL} \delta n} \right)^{2} \cos^{2} \left( {2\pi nX + \varphi } \right)} }}{{1 + \delta \sin \left( {2\pi nX} \right)}}} \right]\Theta^{p + 1} + Q_{gen} \left[ {1 + \chi \Theta } \right] \\ \end{aligned}$$

12 $$S_{BC1} = \Theta (0) - 1.$$

13 $$S_{BC2} = \Theta ^{\prime}(1) - 0.$$

14 $$Loss_{total} = \frac{1}{{N_{col} }}\sum\limits_{i = 1}^{{N_{col} }} {\left\| {S_{ODE} \left( {X_{i} } \right)} \right\|^{2} } + \frac{1}{{N_{col} }}\sum\limits_{i = 1}^{{N_{col} }} {\left\| {S_{BC1} \left( {X_{i} } \right)} \right\|^{2} } + \frac{1}{{N_{col} }}\sum\limits_{i = 1}^{{N_{col} }} {\left\| {S_{BC2} \left( {X_{i} } \right)} \right\|^{2} }$$

Importantly, for the neural network to reliably act as Eq. (11) provides a representation of the ODE, its predictions must strictly comply with the established BCs as denoted in Eqs. (12) and (13), representing a one-dimensional initial thermal equilibrium state, which is typically a critical factor in heat transfer problems. The BCs are uniquely viewed when considering an applied BC at X = 0. The neural network's accuracy concerning that particular BC can be measured using the metric. Each element of the loss function is carefully customized as described in Eq. (14) to estimate the mean square of the error (MSE) throughout the ensemble of locations at which the errors are evaluated. The sum of all loss values, calculated across arbitrary point sets known as collocation points, should converge to zero in the ideal case where a neural network accurately approximates the solution to the heat equation. However, it is acknowledged that combining different loss functions is complex to incorporate into a single cumulative loss function. This difficulty arises from the potential divergence in the magnitudes of these several loss components, which can significantly impact how a model is trained and performs. The neural network may favor a solution that primarily prioritizes the minimization of a dominating loss term when the magnitude of one loss component, or its derivatives, about the model's weights, dramatically surpasses the others. This framework is essential for guaranteeing that all loss terms have comparable magnitudes, which speeds up the simultaneous minimization of all losses during the training stage.

## Results and discussion

The thermal dispersal in a wavy fin design exposed to convective impacts and internal heat generation is examined in this research. As an aspect of machine learning's sophisticated approaches to analyzing heat transmission in a convective wavy fin, this investigation's innovative operation of a physics-informed neural network (PINN) elucidates the thermal distribution. The current research extends the implementation of PINNs to investigate the implications of the temperature equation's nonlinearity and boundary conditions by adjusting the architecture's hyperparameters. Using the non-dimensional components, the non-linear ODE referring to heat conduction is simplified into a dimensionless version. The following section comprises a graphical and tabular report of the thermal variance in the convective wavy fin.

**Figure 3:**
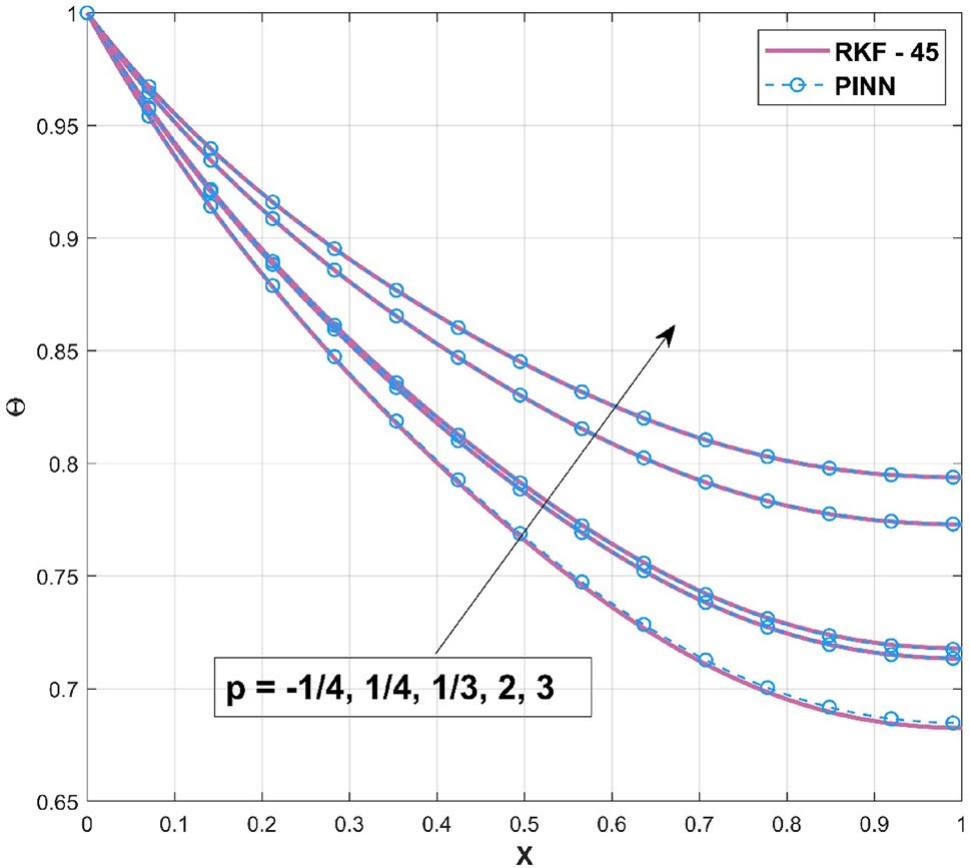
Comparison of PINN and RKF-45 results.

**Figure 4:**
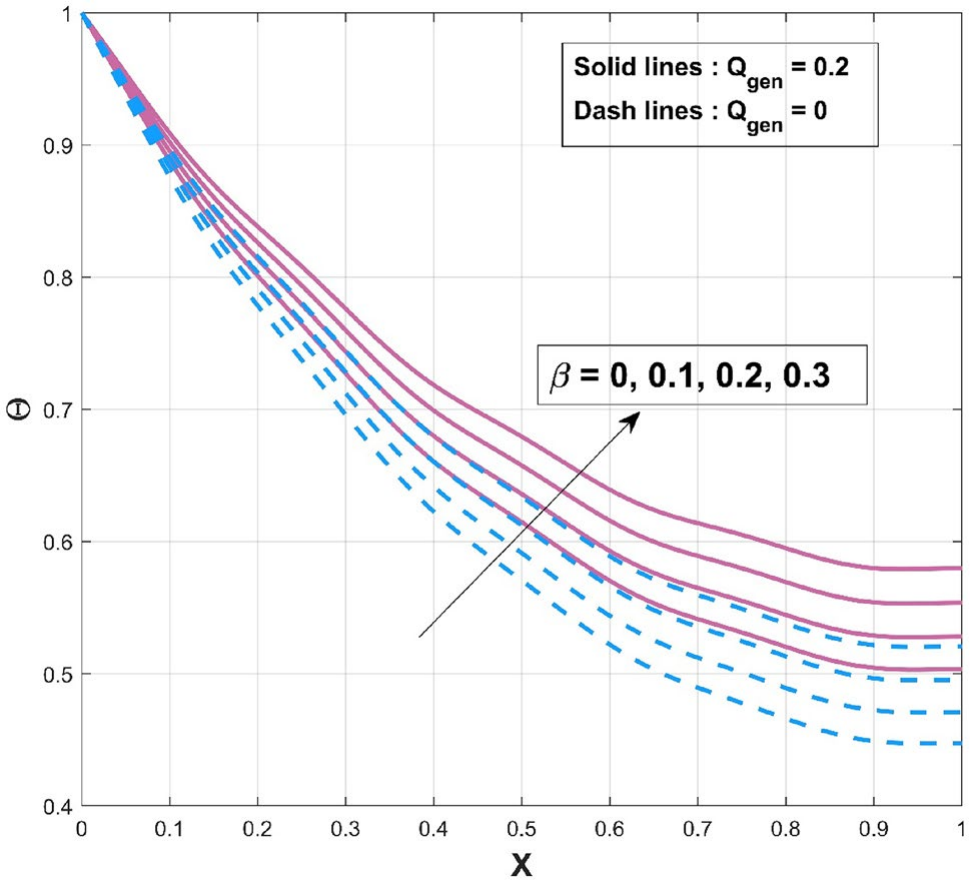
Influence of $\beta$ on $\Theta$ of WFRS.

**Figure 5:**
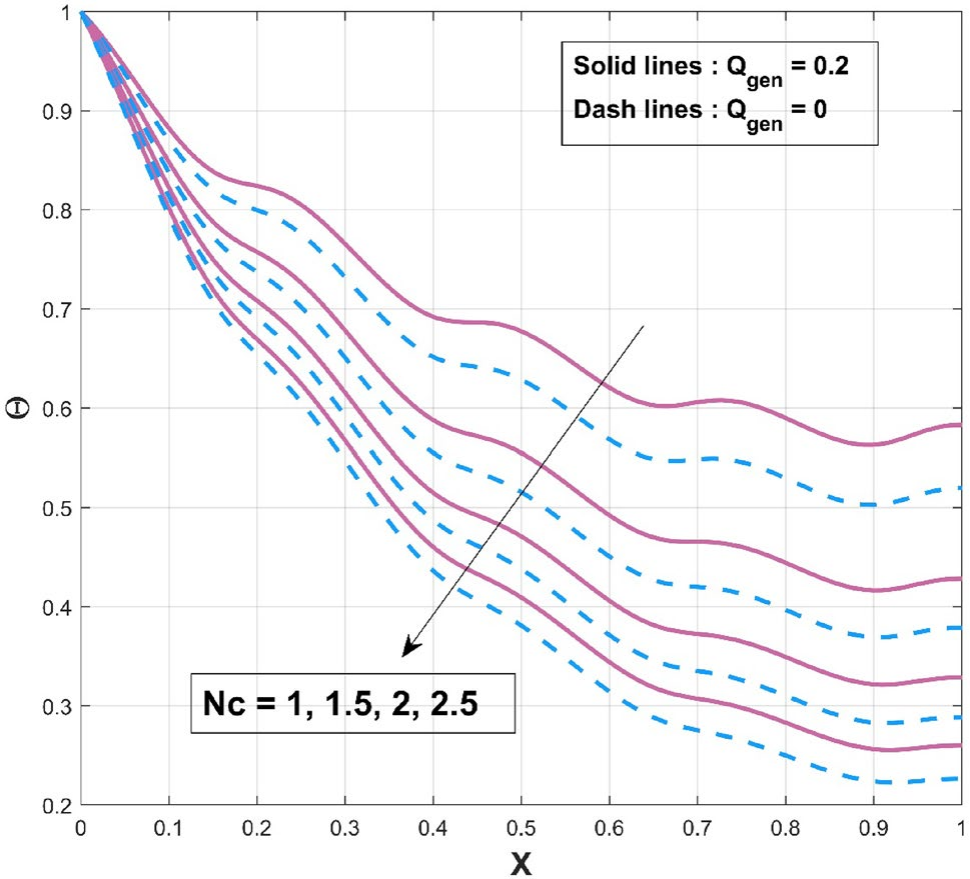
Influence of $Nc$ on $\Theta$ of WFRS.

**Figure 6:**
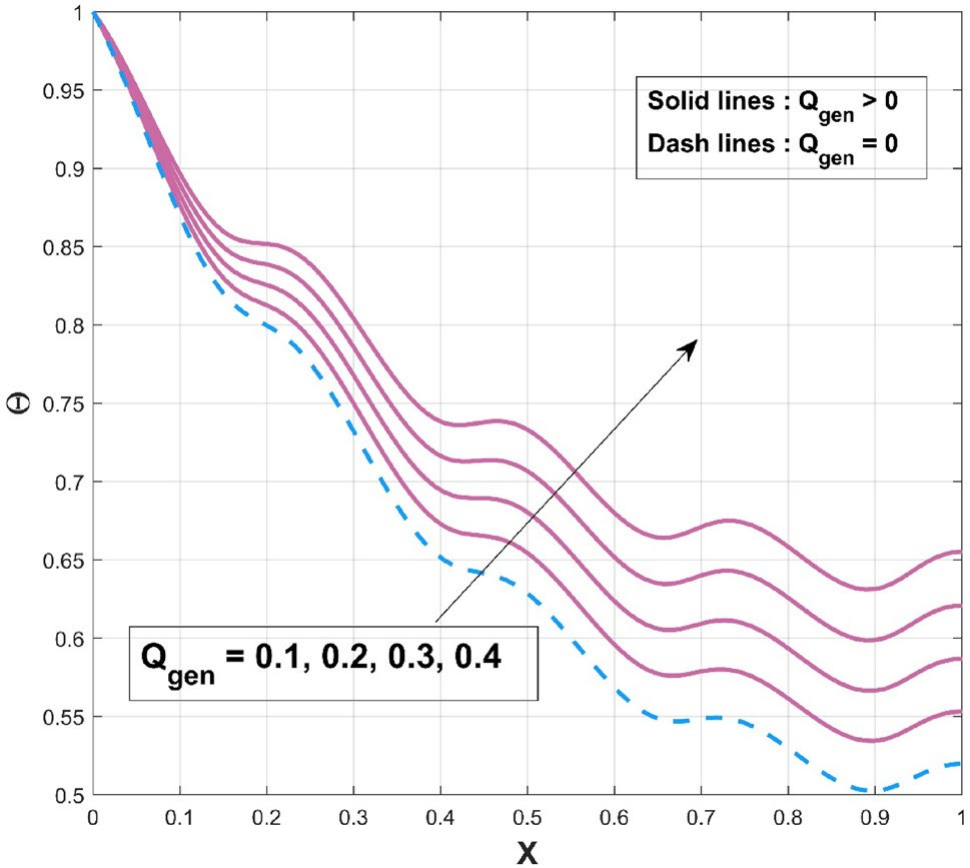
Influence of $Q_{gen}$ on $\Theta$ of WFRS.

**Figure 7:**
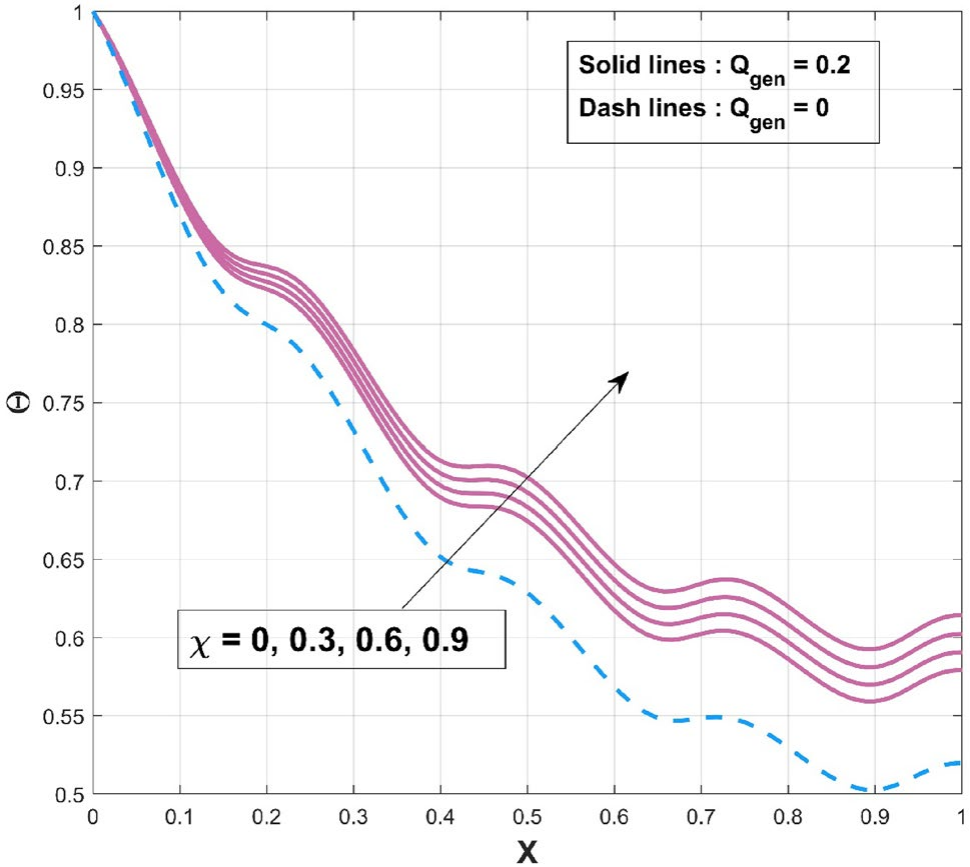
Influence of $\chi$ on $\Theta$ of WFRS.

This study developed an innovative solution based on the combination of physics and machine learning to tackle the challenges encountered in solving the heat transfer equation (ODE) in the presence of internal heat generation. The foundation of this strategy is to precisely plan the training of a neural network to optimize a comprehensive loss function. This loss function is carefully built to satisfy the ODE, and BC simultaneously, ensuring a comprehensive and accurate solution. Different features are included by using the principles of the heat transfer problem to infuse the neural network with physics-based insights. This solution addressed the varied magnitudes of loss terms, which improved the network's robustness and efficiency. Furthermore, the training sites are chosen purposefully to increase the concentration of data points around discontinuities in the loss function and input parameters. While training PINNs to solve differential equations (DEs), selecting activation functions is an important consideration. The vanishing gradients can cause problems with conventional activation functions like sigmoid or hyperbolic tangent (tanh), which might restrict the network's learning ability. In contrast, the Rectified Linear Unit (ReLU) function is frequently used in various deep learning applications because it successfully solves the vanishing gradient issue. Calculating the loss function for DEs like heat transfer equations requires precisely computing the first and second derivatives of the neural network concerning its inputs. Previously, the difficulty provided by the various loss values, their magnitudes, and their effect on training is noted. Using an adaptive normalization approach can help to solve this problem. The Glorot uniform initializer is primarily used to initialize the model, which causes the loss terms' magnitudes to vary. Due to the dominance of more extensive error terms in the training, more minor magnitude error terms may be more challenging to reduce. Loss terms are carefully defined to maintain comparability of the individual error terms as the model develops. The adaptive normalization system was created for every 50 epochs on average to update normalization factors. The new normalization factors are calculated based on the relationship between a specific loss and the most significant loss term. If this ratio is greater than a preset cutoff, such as 0.000001, the normalization factor for the particular loss term is changed to unity. However, the balance falls below the threshold, indicating that the loss term is significantly lower than the most significant loss term, and the normalization factor is modified to the loss ratio divided by the threshold. With this modification, the relative error term is below the epoch's threshold. The normalization factor will get stronger during the subsequent updated interval if the most significant loss term evolves more slowly during training than the specific loss term. This tactical approach directs the model towards a solution for the loss term with the largest magnitude with a minor influence from other loss terms. Keeping this in mind, Fig. 3 is drawn to indicate the accuracy of the applied PINN technique by comparing it with the numerical methodology (RKF-45). The comparison is specifically carried out for all $p$ $\left( {p = - \frac{1}{4},\frac{1}{4},\frac{1}{3},2,3} \right)$ scenarios that are taken into consideration. It is worth mentioning to highlight that the acquired outcomes are excellently in accordance with the RKF-45 results and are accordingly extremely equivalent to the findings. Additionally, comparative analysis confirms the validity of the findings. The influence of $\beta$ on the temperature variation of the internal heat generating wavy fin is revealed in Fig. 4. It is noted that, increase of temperature curves is caused with an augmented in thermal conductivity constraint. The heat within the fin's structure is elevated as an effect of improved heat conduction from the fin base caused by amplification in its thermal conductivity gradient. The quantity of heat transferred by conduction to heat discharge by convection is identified as $Nc$. Convective heat loss impact described by the parameter $Nc$ on the temperature variation of the wavy fin is discussed through plot as divulged in Fig. 5. Rise in the convective-conductive variable deceases the thermal distribution in the fin. The distribution curve exhibits the highest temperature gradient at the lowest $Nc$ value, while its significantly higher magnitude is caused by the thermal conductivity value, which is considerably lesser than the remaining variables. Moreover, the effectiveness of convective heat transfer within the fin improves as $Nc$ elevates owing to the overall temperature in the fin dropping more rapidly. As the heat transmitted by convection at the base improves, the temperature drops along the fin, particularly at the tip. It follows that as fin convective heat exchange rises, a greater amount of heat is conducted across the fin, which raises the operating temperature dispersion in the fin and, in turn, the heat transfer rate. Significant consequence of the internal heat generation parameter on the thermal dispersal of the fin is denoted in Fig. 6. Thermal variation increases with an upsurge of this parameter. Precisely, convective wavy fin without heat generation ($Q_{gen} = 0$) is illustrated by the bottom curve. When the heat generation ($Q_{gen} = 0.1,\,\,0.2,\,\,0.3,\,\,0.4$) rises, the local fin temperature rises as well denoting the existence of strong internal heat generation. In other words, the diminution of the heat generating characteristic results in a temperature profile decrease, signifying increased heat loss along the fin surface. Figure 7 explicates the variation in temperature as a factor of heat generating coefficient. The upsurge in the thermal distribution is due to the rise of this variables. The temperature gradient progressively develops by altering the heat generation variable. However, more heat production culminates in increased fin temperatures because, in a steady state, an increased quantity of heat needs to be transferred into the environment through the fin. The increased internal heat production causes the fin to dissipate more heat into its surrounding atmosphere, resulting in improved dimensionless fin temperature.

**Figure 8:**
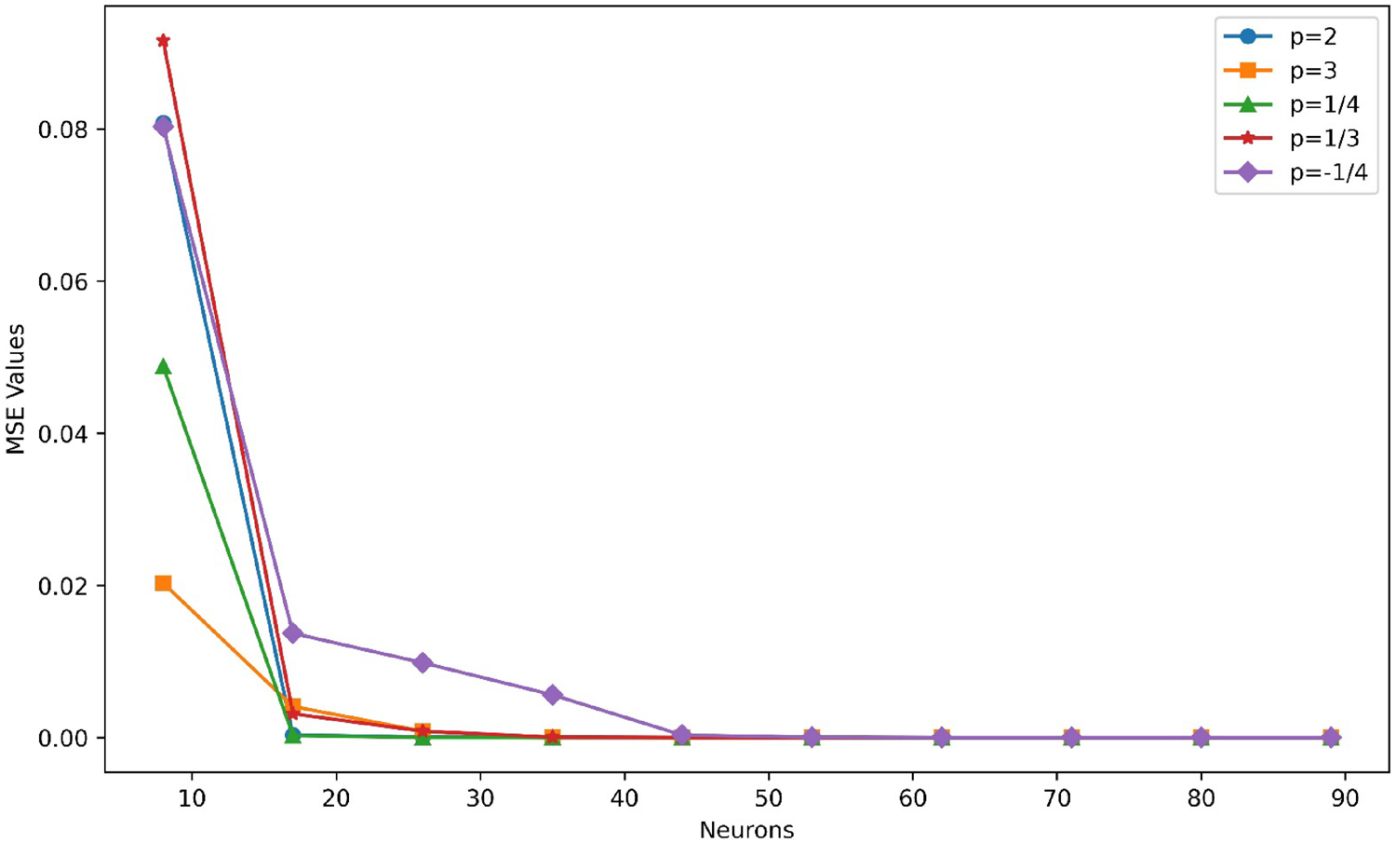
MSE results of the PINN training for different neurons.

**Figure 9:**
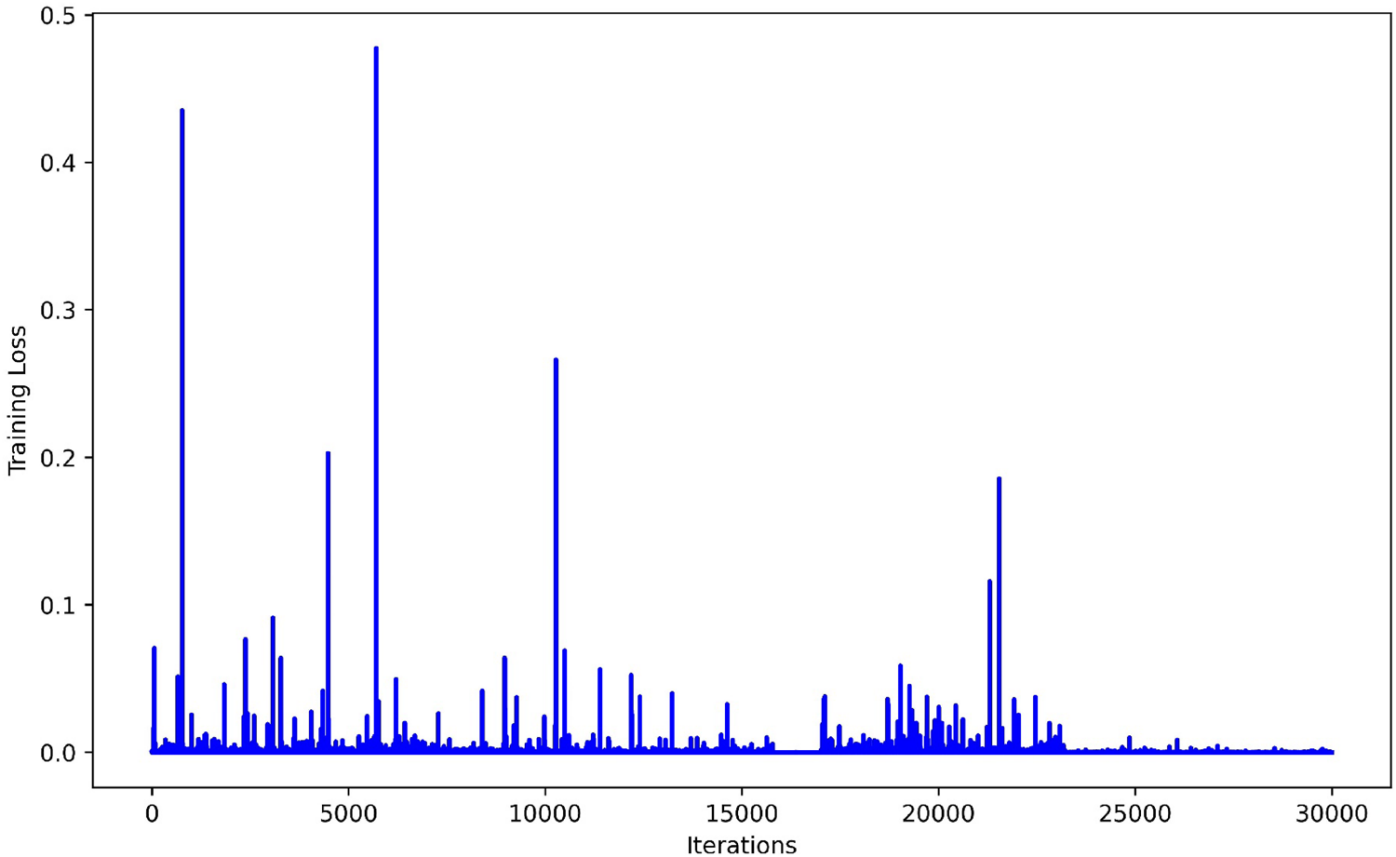
Training loss of the PINN training.

**Figure 10:**
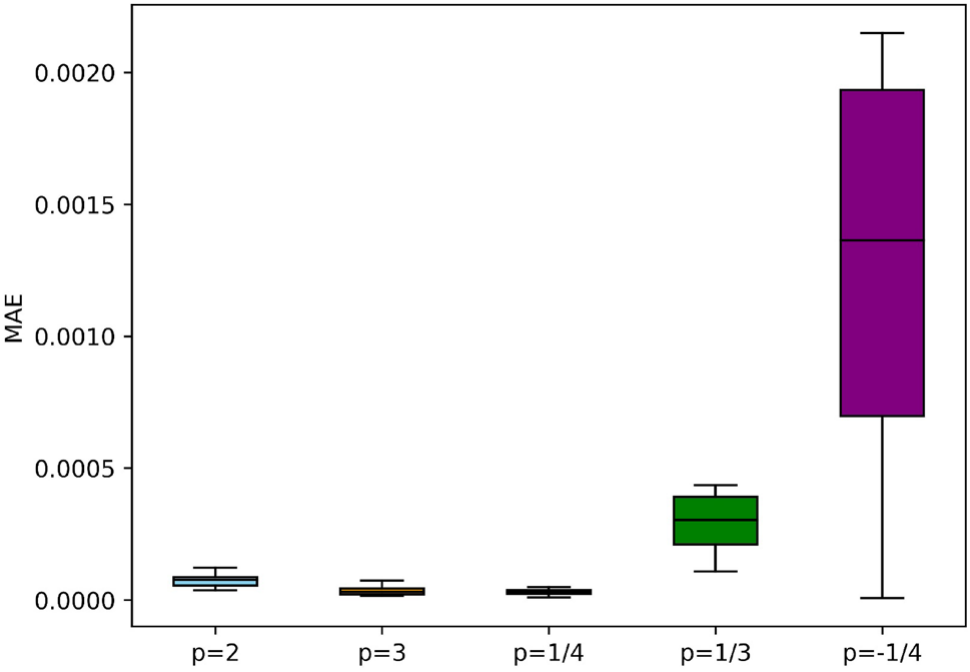
Error plot for MAE results of the PINN training.

The examination of mean squared error (MSE) values for different neuron topologies provides insight into the connection between prediction performance and model complexity. A comparison analysis using different network units is shown in Fig. 8. The other hyperparameters, namely the learning rate of 1E−04 and the epochs set to 30,000, are fixed. The analysis is noteworthy since it includes two neuron layers and shows a continuous pattern of lowering MSE values for all cases as the number of neurons increases from 8 to 89. This pattern suggests that a greater neuronal density enhances the network's capacity to represent and approximate the underlying physics, leading to more precise predictions. The MSE is the preferred loss function, and convergence is seen at 62 neurons in the figure, with errors ranging from 1.01E−09 to 2.06E−06. These findings highlight the potential advantages of using more complex neural network architectures in the context of physics-informed modeling, emphasizing the significance of carefully selecting and modifying the neural network structure to obtain optimal prediction accuracy. A graph of iterations against training loss is shown in Fig. 9, which offers insightful detail about the convergence performance of the PINN technique. An obvious trend shows a progressive decrease in the training loss as the number of epochs increases. Notably, the plot shows a clear convergence point of 30,000 iterations, indicating that the model has reached a stable state where additional iterations do not significantly contribute to the decrease of training loss. This convergence phenomenon is critical in understanding the PINN's training dynamics since it indicates that the model has sufficiently learned the underlying physics and no longer undergoing substantial adjustments. The error box plots in Fig. 10 analyses the absolute errors for the five distinct cases and provides a detailed perspective on the model's predictive accuracy. Each box represents the distribution of absolute errors, the median absolute error and interquartile range vary for each case, reflecting the dispersion of errors in the predictions. For instance, in the first three cases, the box plot demonstrates a relatively narrow distribution between 1.22E−04 to 9.90E−06, indicating consistent and accurate predictions. The errors range between 2.15E−03 and 7.63E−06 while the *p*-value is − 1/4 exhibits a wider spread, suggesting a higher variability in absolute errors. Identifying such variations in error distribution is crucial for understanding the robustness and reliability of the physics-informed neural network across different scenarios. Thus, by the box plot evaluation it is observed that the errors are in considerable range for all cases.

In Table 1, numerical findings for the thermal distribution based on various index of convective process parameter $\left( {p = - \frac{1}{4},\frac{1}{4},\frac{1}{3},2,3} \right)$ values are reported. The thermal values decrease along the fin length and an upsurge in these values are observed with an increment in $p$. This table shows that the method used is extremely convergent, and the results are accurate to maximum of four or five decimal places. Also, Table 2 provides the validation of the performed PINN calculations with the published work of Khaled^54^. The result of the PINN excellently converges with the compared outcomes.

**Table 1:** Validation of the PINN results against RKF-45 findings.

${\text{p}}$	${\text{X}}$	$\Theta$		
		PINN	RKF-45	Error
−1/4	0	1.000007	1.000000	7.62939E−06
	0.2	0.883129	0.883691	0.000561046
	0.4	0.798810	0.799922	0.001111697
	0.6	0.734608	0.736249	0.001640231
	0.8	0.694266	0.696286	0.002019562
	1	0.682647	0.684796	0.002148474
1/4	0	0.999951	1.000000	4.82798E−05
	0.2	0.892658	0.892695	3.66471E−05
	0.4	0.816564	0.816588	2.36785E−05
	0.6	0.759252	0.759288	3.56306E−05
	0.8	0.723614	0.723627	1.23073E−05
	1	0.713446	0.71347	2.37405E−05
1/3	0	1.000108	1.000000	0.000108004
	0.2	0.894164	0.893973	0.000191383
	0.4	0.819222	0.818943	0.000279093
	0.6	0.762864	0.76253	0.000334411
	0.8	0.727856	0.727461	0.000395874
	1	0.717921	0.717487	0.000434257
2	0	0.999963	1.000000	3.61204E−05
	0.2	0.912096	0.912148	5.16784E−05
	0.4	0.852048	0.85211	6.13645E−05
	0.6	0.807671	0.807757	8.52146E−05
	0.8	0.780521	0.780621	9.9786E−05
	1	0.772955	0.773073	0.000117642
3	0	0.999983	1.000000	1.62721E−05
	0.2	0.919179	0.919204	2.42028E−05
	0.4	0.864789	0.864811	2.16333E−05
	0.6	0.824819	0.824861	4.14948E−05
	0.8	0.800506	0.800555	4.8289E−05
	1	0.793797	0.793862	6.44474E−05

**Table 2:** Comparison of PINN outcomes against work of Khaled^54^ for $\beta = 0$, $a_{RL} = 0$,$p = 2$, $Q_{gen} = 0$, and $\delta = 0$.

$Nc$		0.25	1.00
$\Theta \left( 1 \right)$	Khaled^54^	0.886819	0.648054
	PINN	0.886754	0.648013
	Error	6.5E−5	4.1E−5

## Conclusion

The present investigation adopts a convective effect to study the thermal response of a wavy fin under the influence of internal heat generation. The dimensionless terms are implemented to simplify the formulated equation, which is solved by two approaches. Numerically using RKF-45 and, through deep learning by the proposed PINN model. The following conclusions are drawn from the proposed analysis:

- An increase in the thermal conductivity variable upsurges the temperature variation in the wavy fin.
- A decrease in the thermal variation is observed for an augmentation in the convective-conductive parameter.
- Elevated scales of internal heat generation parameter promote thermal dispersal in the wavy profiled fin.
- The thermal profile of the wavy fin proliferates with an escalation in the index of the convective process parameter.
- According to the findings, the PINN displayed commendable alignment with numerical results in the training. The PINN, enhanced with designed characteristics, excelled at capturing the problem's underlying physics. As a result, it outperformed typical neural networks in its capacity to make exact predictions outside of the training environment.
- The characteristics of traditional physics-based modelling are combined with the adaptability and learning capabilities of the PINN. It provides a precise and effective solution, decreasing the requirement for enormous data sets and minimizing the computational costs while retaining high accuracy by explicitly embedding the governing equations into the neural network architecture.

The present investigation also proposes various kinds of prospective study possibilities for designing wavy fins and their practical applications. A persistent endeavour remains essential to identify the optimal thermophysical properties and the conditions that may facilitate heat transport from the hot body to its surroundings.

corresponding author upon reasonable request.

## Data Availability

The datasets used and/or analyzed during the current study are available from the corresponding author upon reasonable request.

## List of symbols

*L*: Fin’s length
$x$: Fin axial distance
$$p$$: Exponent constant
$a_{RL}$: Fin profile aspect ratio
$Nc$: Convection-conduction parameter
$X$: Fin’s length (dimensionless)
$\delta$: Surface wave dimensionless amplitude
$Q_{gen}$: Internal heat generation parameter (dimensionless)
$\varphi$: Surface wave phase shift
$$T_{a}$$: Ambient temperature
$\beta$: Thermal conductivity parameter
$\chi$: Internal heat generating rate (dimensionless)
$L_{ta}$: Wavy fin total arc length
$\nu$: Parameter of heat generation
$T_{b}$: Temperature at the fin’s base
$H_{0}$: Fin base half height
$$q^{*} (T)$$: Heat generation
$$n$$: Wave number
${\rm A}_{SF}$: Fin surface area
$\Theta$: Non-dimensional temperature
$${\rm K}$$: Thermal conductivity
$H$: Fin half height
$h^{*}$: Convective heat transfer coefficient
${\rm A}_{CS}$: Fin cross-sectional area
$$T$$: Temperature
$$W$$: Fin’s width
$\vartheta$: Thermal conductivity variation parameter

## Subscript

$$a$$: Ambient
$$b$$: Base
